# Sulforaphane-cysteine downregulates CDK4 /CDK6 and inhibits tubulin polymerization contributing to cell cycle arrest and apoptosis in human glioblastoma cells

**DOI:** 10.18632/aging.103537

**Published:** 2020-08-29

**Authors:** Juntao Li, Yan Zhou, Yuting Yan, Zhongnan Zheng, Yabin Hu, Wei Wu

**Affiliations:** 1 Department of Biochemistry and Molecular Biology, School of Basic Medical Sciences, Capital Medical University, Beijing, China; 2 Beijing Key Laboratory for Tumor Invasion and Metastasis, Capital Medical University, Beijing, China

**Keywords:** glioblastoma, CDK4, CDK6, microtubule, cell cycle

## Abstract

Here we demonstrated that sulforaphane-cysteine (SFN-Cys) regulated cell cycle-related protein expressions in G0/G1 and G2/M phases of U87MG cells via High Performance Liquid Chromatography-Mass Spectrometry/Mass Spectrometry (HPLC-MS/MS) and proteomics analysis. Further, mRNA products of CDK4, CDK6 and α-tubulin were significantly higher in glioblastoma than those in normal tissues, and these results were significantly correlated to pathological grades and clinical prognosis via analyzing TCGA and CGGA databases. Furthermore, Western blot showed that SFN-Cys downregulated CDK4, CDK6 and p-Rb in a dose-dependent manner and these results were reversed by p-ERK1/2 blocker PD98059 in U87MG and U373MG cells. The reductions of CDK4, CDK6 and p-Rb were reversed by proteasome inhibitor MG132; similarly, the upregulation of 26S proteasome by SFN-Cys was reversed by PD98059. Interestingly, SFN-Cys decreased CDK4 and CDK6 by phosphorylated ERK1/2-caused proteasomal degradation resulting in decreased Rb phosphorylation contributing to cell cycle arrest in G0/G1 phase. Besides, Western blot showed that SFN-Cys downregulated α-tubulin resulting in microtubule disruption and aggregation, and cell cycle arrest in G2/M phase and apoptosis. These results might help us understand the molecular etiology of glioblastoma progression to establish brand-new anti-cancer therapies.

## INTRODUCTION

Brain glioblastoma (GBM) is a fatal tumor causing low survival and poor prognosis [1–3]. Due to the highly invasive feature, the primary tumor was not able to be removed by surgery thoroughly. Therefore, it is essential to establish more powerful chemotherapies. We found that sulforaphane (SFN) and its analog SFN-Cys induced apoptosis in GBM [4, 5]. However, we need to find out the working mechanisms that SFN or SFN-Cys inhibits tumors so that we may determine the potential molecule targets.

Cell cycle progression was regarded to affect cell proliferation, migration and invasion. Cell cycle was controlled by cell cycle checkpoints which prevented cells from entering the next phase of cell cycle [6]. A critical checkpoint occurs in G1 phase and controls the entry of cells into S phase and the start of DNA synthesis. Cyclin-dependent kinases 4 and 6 (CDK4 and CDK6) are the key enzymes for cells to enter S phase from G1 phase [7]. Protein Rb was phosphorylated by the activated Cyclin D-CDK4/CDK6 complex leading to the release of transcription factor E2F to nucleus to initiate DNA replication and make the cells enter S phase [8]. CDK4 and CDK6 were highly expressed in many tumor cells [9–12]. The activity of Cyclin D-CDK4/CDK6 complex was negatively regulated by P16 coded by CDKN2A and CDKN2B. The gene deletion of CDKN2A and CDKN2B in tumor cells resulted in high activities of CDK4/CDK6, and made cells transit from G1 phase to S phase leading to rapid cell proliferation [13, 14]. Other studies showed that CDK4 and CDK6 played a role in anti-apoptosis via inhibiting the activation of Caspase 3 in tumor cells [15]. High phosphorylation of Rb by activating Cyclin D-CDK4 /CDK6 complex was an important reason for the rapid proliferation in GBM cells [16] and the proliferation capacity of tumor cells was significantly reduced after the intervention of CDK4/CDK6 inhibitors [17]. Hence, it is possible to design efficient anti-tumor drugs to treat GBM patients.

It has been reported that SFN downregulated oncoproteins via proteasome-dependent mechanism [18–20]. More, we found that SFN and its analogs took effect by phosphorylating ERK1/2 activating the proteasome system to degrade a variety of tumor-related proteins, such as microtubule proteins [20–22]. Studies showed that CDK4/CDK6 were mainly degraded by the ubiquitin-proteasome pathway [23–26]. Other studies showed that SFN downregulated CDK4/CDK6 in breast and ovarian cancer cells leading to cell cycle arrest in G0/G1 phase and apoptosis [27–29]. Therefore, we speculated that SFN-Cys might cause cell cycle arrest in G0/G1 phase in GBM cells.

Another important protein for cell mitosis is α-tubulin. Studies showed that microtubule was an important target in the treatment of glioblastoma; microtubule disruption significantly caused apoptosis [30–32]. We previously reported that SFN-Cys suppressed proliferation and induced apoptosis via disrupting microtubules in human prostate cancer and non-small cell lung cancer [21, 22]. Therefore, SFN-Cys might induce cell cycle arrest in G2/M phase and apoptosis resulting from microtubule disruption in GBM cells.

Taken together, SFN-Cys might activate proteasome downregulating oncoproteins in cell cycle checkpoints leading to cell cycle arrest and apoptosis. These studies will help us further understand the mechanisms that SFN-Cys inhibits cell growth to establish new and efficient anti-GBM therapies.

## RESULTS

### The levels of CDK4/CDK6 and TUBA1C are the important indicators for clinicopathological grades and patient prognosis

**Figure 1:**
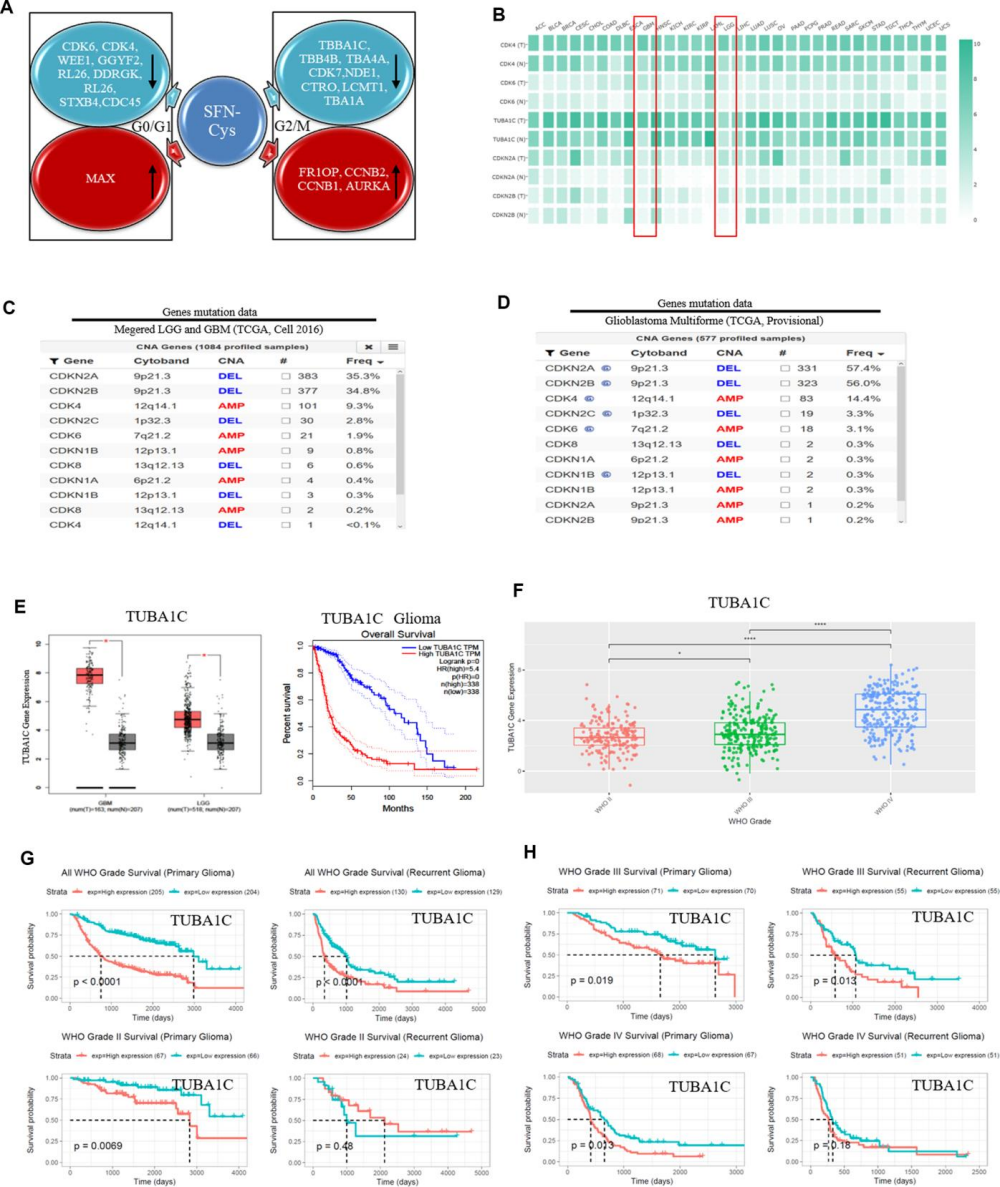
**SFN-Cys decreased the expressions of CDK4/CDK6 and TUBA1C.** TUBA1C mRNA expression showed positive correlation to pathological grades and negative correlation to clinical prognosis in glioma. The deletion rates of CDKN2A and CDKN2B were higher in glioma patients. (**A**) After U87MG cells were treated with or without SFN-Cys for 24 h, the extracted proteins were analyzed for expression; protein expressions and Ontology were analyzed via HPLC-MS/MS and the Uniprot net. (**B**) Differential expressions of mRNA were analyzed in normal and tumor tissues via GEPIA database, which matched TCGA normal and GTEx data [46]. LGG: Low grade glilma. (**C**, **D**) The deletion rates of CDKN2A and CDKN2B were analyzed by means of database of Glioblastoma Multiforme (TCGA, Provisional) and of Merged Cohort of LGG and GBM (TCGA, Cell 2016) via cBioPortal for Cancer Genomics. (**E**) The expression and survival analysis of TUBA1C mRNA in glioma patients were done via GEPIA Database, which matched TCGA normal and GTEx data. (**F**–**H**). The expression and survival analysis of TUBA1C mRNA in glioma patients with primary and recurrent were done via CGGA database. Data were shown as *P < 0.05; **P < 0.01; ***P < 0.001, NS no significance vs. control group, n ≥ 3.

**Figure 2:**
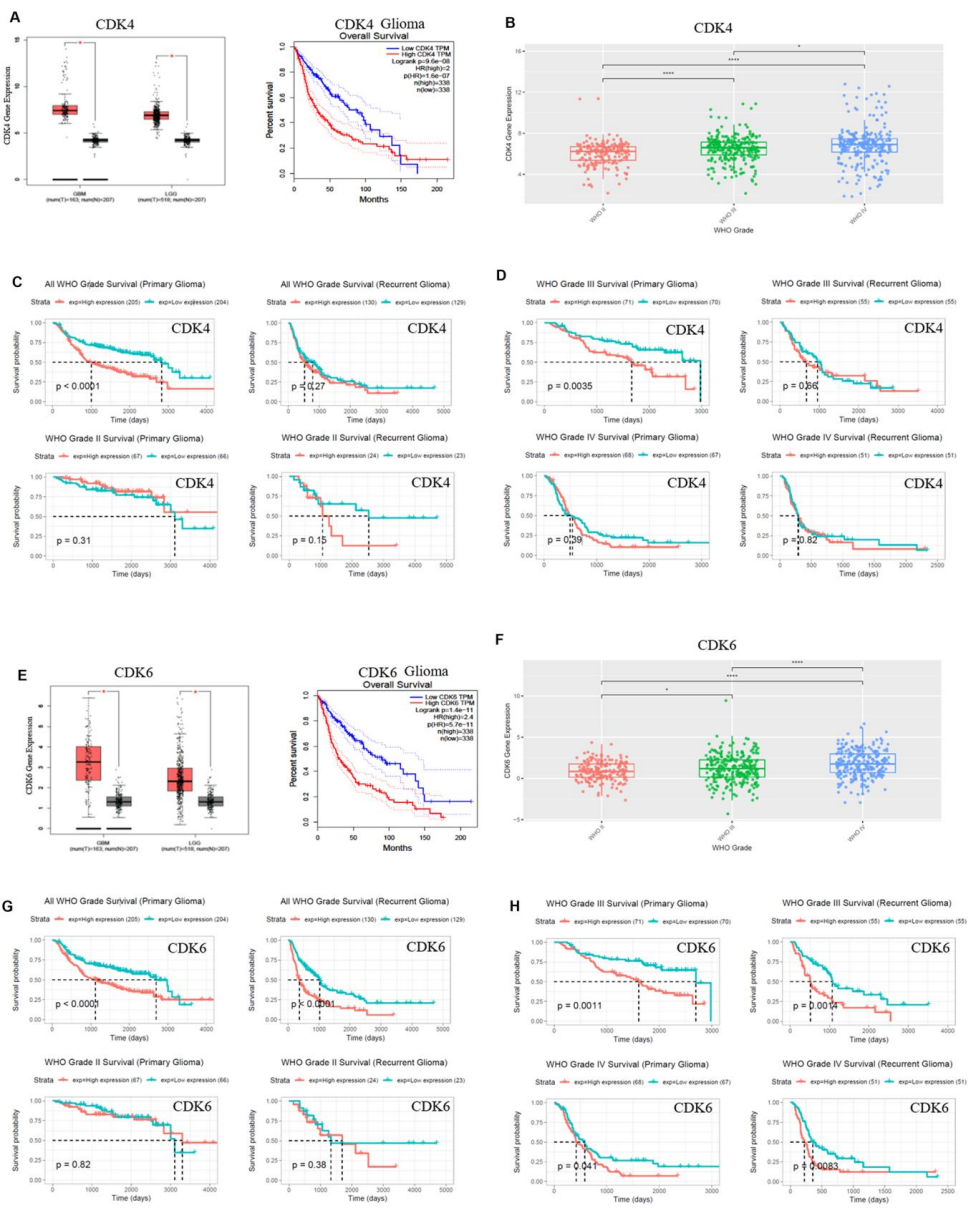
**CDK4/CDK6 mRNA expression showed positive correlation to pathological grades and negative correlation to clinical prognosis.** (**A**, **E**) The expression and survival analysis of mRNA CDK4/CDK6 in glioma patients were done via GEPIA Database, which matched TCGA normal and GTEx data. (**B**–**D**, **F**–**H**). The expression and survival analysis of mRNA CDK4/CDK6 in glioma patients with primary and recurrent were done via CGGA database. Data were shown as *P < 0.05; **P < 0.01; ***P < 0.001, NS no significance vs. control group, n ≥ 3.

We recognized the significantly differential expressions of cell cycle-related proteins in response to SFN-Cys, and they were mainly expressed in G0/G1 and G2/M phases via HPLC-MS/MS and gene ontology analysis (Figure 1A). Further, we found that three mRNA expressions were closely related to the pathological grades and clinical prognosis in glioblastoma patients via the analysis of TCGA (The Cancer Genome Atlas) and CGGA (the Chinese Glioma Genome Atlas) databases, respectively with expressions of CDK4/CDK6 in G0/G1 phase and TUBA1C in G2/M phase (Figure 1E–1H), (Figure 2A–1H). The CDK4/CDK6 and TUBA1C mRNA levels were higher in glioblastomas than those in normal brain tissues (Figure 1B).

CDK4/CDK6, TUBA1C expressions in 693 samples with mRNA microarray data were analyzed according to the clinicopathological grades in CGGA cohort. CDK4/CDK6, TUBA1C mRNA expressions in grade IV were higher than those in grades II and III (Figure 1F), (Figure 2B, 2F). These results were confirmed in TCGA RNA sequencing data (Figure 1E), (Figure 2A, 2E), which further suggested that higher expressions of CDK4/CDK6, TUBA1C were accompanied by higher malignancy in glioblastomas.

Survival data were available for 693 patients in CGGA and 676 patients in TCGA database, respectively. Survival rates of patients with higher CDK4/CDK6, TUBA1C expressions were significantly shorter than those of the counterparts (Figures 1E, 1G, 2A, 2C, 2E and 2G). Due to heterogeneity of glioma, we additionally investigated the patient prognosis with CDK4/CDK6 and TUBA1C expression in every grade of glioma in CGGA datasets. Results showed that patients with higher TUBA1C expression had shorter survival rates than those of the counterparts in grades II, III and IV primary glioma and grade III recurrent glioma, but not in grades II and IV recurrent glioma (Figure 1G, 1H). The patients with higher CDK4 expression had shorter survival rate than those of the counterparts in grade III primary glioma, but not in grades II and IV (Figure 1C, 1D). The patients with higher CDK6 expression had shorter survival rates than those of the counterparts in grades III and IV primary glioma and recurrent glioma, but not in stage II (Figure 2G, 2H). These findings indicated that TUBA1C and CDK4/CDK6 were important indicators for clinicopathological grades and prognosis of patients.

### Deletions of CDKN2A and CDKN2B were shown widely in GBM

We analyzed the gene deletion in glioma via cBioPortal net with the Glioblastoma Multiforme (TCGA, Provisional) and Merged Cohort of LGG and GBM (TCGA, Cell 2016) databases, respectively. With the analysis of database Merged Cohort of LGG and GBM (TCGA, Cell 2016), we found that the rate of CDKN2A locus 9p21.3 deletion was up to 35.3% in GBM, and the rate of CDKN2B locus 9p21.3 deletion was up to 34.8%. The rate of CDK4 locus 12q14.1 mutated amplification was 9.3%, and the rate of CDK6 locus 7q21.2 mutated amplification was 1.9%. (Figure 1C). With the analysis of database Glioblastoma Multiforme (TCGA, Provisional), we found that the deletion rate of CDKN2A locus 9p21.3 was up to 57.4%, and the deletion rate of CDKN2B locus 9p21.3 was up to 56%. The rate of CDK4 locus 12q14.1 mutated amplification was 14.4%, and the rate of CDK6 locus 7q21.2 mutated amplification was 3.1% (Figure 1D).

### SFN-Cys decreased the expression of p-Rb, CDK4 and CDK6 via ERK1/2 phosphorylation

**Figure 3:**
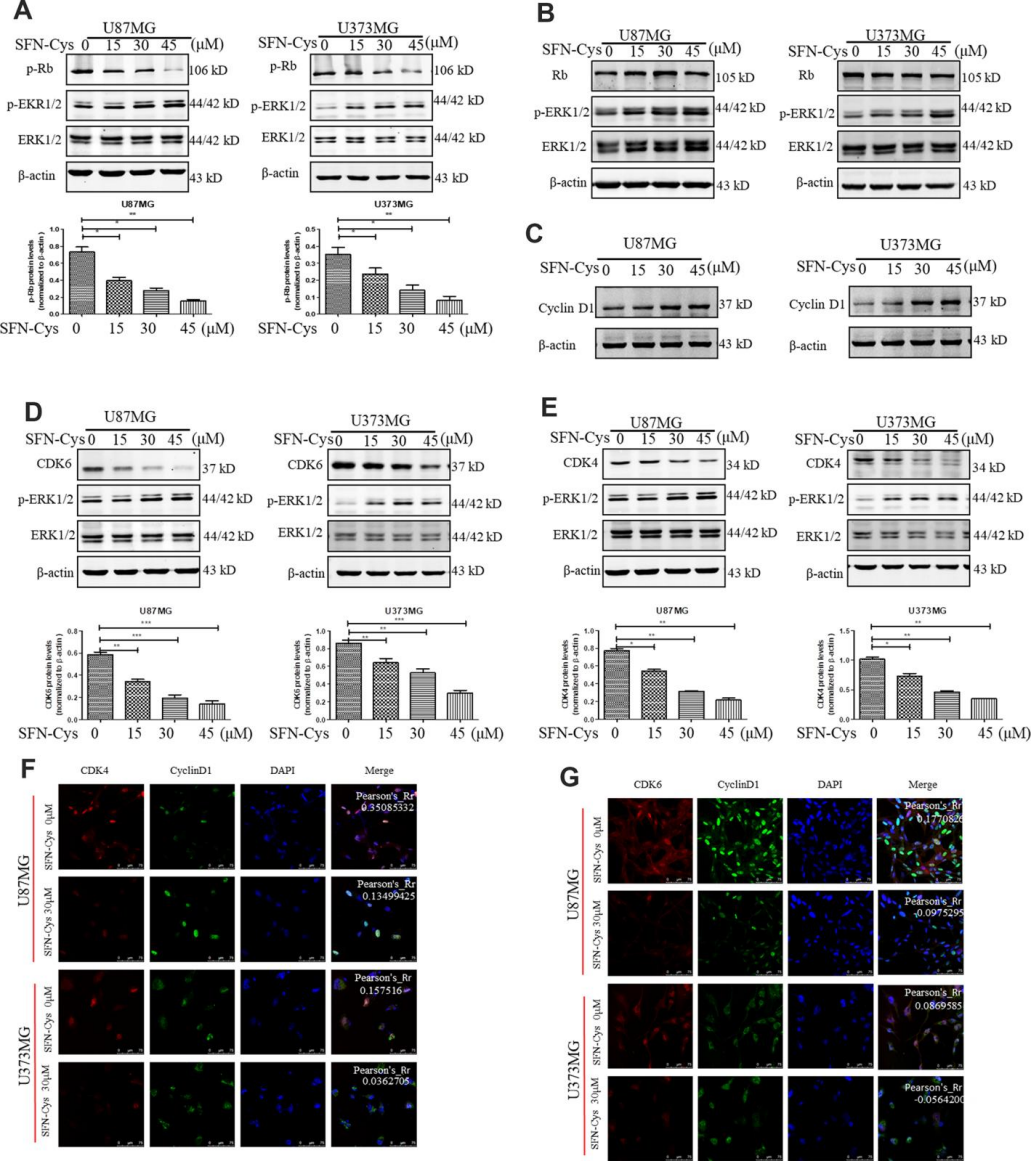
**SFN-Cys decreased the expressions of p-Rb, CDK4 and CDK6; while increased the expressions of p-ERK1/2 and Cyclin D1, and decreased the co-localization of CDK4, CDK6 to Cyclin D1.** (**A**–**E**): Western blot showed the expressions of p-Rb, Rb, CDK4, CDK6, p-ERK, and Cyclin D1 in both U87MG and U373MG cells treated with SFN-Cys at the indicated concentrations. (**F**, **G**) Immunofluorescence and confocal microscopy were employed to observe the co-localization of CDK4, CDK6 to Cyclin D1 in both U87MG and U373MG cells. Scale bar: 75 μm. Data were shown as means ± SD (n ≥ 3). *, P ≤ 0.01.

**Figure 4:**
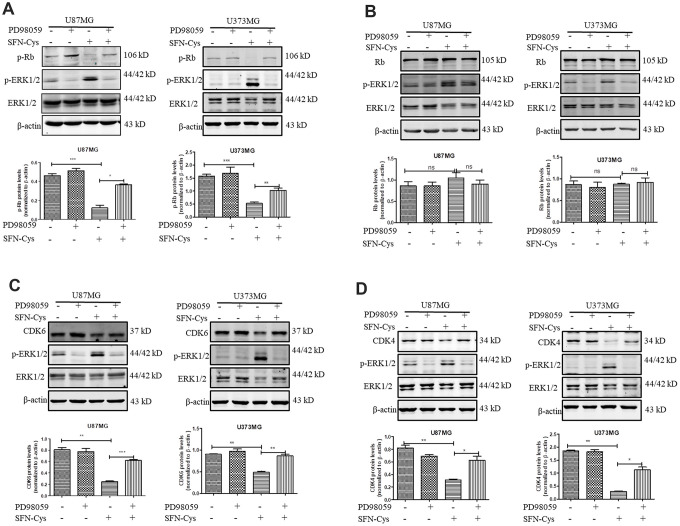
**SFN-Cys decreased the expressions of CDK4, CDK6 and p-Rb via sustained ERK1/2 phosphorylation.** (**A**–**D**) Both U87MG and U373MG cells were pretreated with PD98059 (25 μM) for 30 min, then treated with SFN-Cys (30 μM) for 24 h. The expressions of CDK4, CDK6, p-Rb, Rb and pERK1/2 were analyzed by Western blot. Data were shown as means ± SD (n ≥ 3). *, P ≤ 0.01.

Western blot was used to verify the effect of SFN-Cys on expression of Cyclin D1, CDK4/CDK6, p-Rb and Rb. We found that SFN-Cys significantly downregulated the expressions of CDK4/CDK6 and p-Rb, while significantly upregulated the expression of p-ERK1/2 in a dose-dependent manner in U87MG/U373MG cells and the expression of Rb was not affected (Figure 3A, 3B, 3D and 3E). Although the expression of Cyclin D1 was increased with the intervention of SFN-Cys, the co-localizations of CDK4/CDK6 to Cyclin D1 were decreased (Figure 3C, 3F and 3G). We further found that p-ERK1/2 inhibitor PD98059 reversed the CDK4/CDK6 and p-Rb downregulation induced by SFN-Cys, indicating that SFN-Cys downregulated the expressions of CDK4/CDK6 and p-Rb via ERK1/2 activation (Figure 4A–4D).

### SFN-Cys downregulated CDK4/CDK6 via activating 26S proteasome inducing apoptosis by ERK1/2 phosphorylation

**Figure 5:**
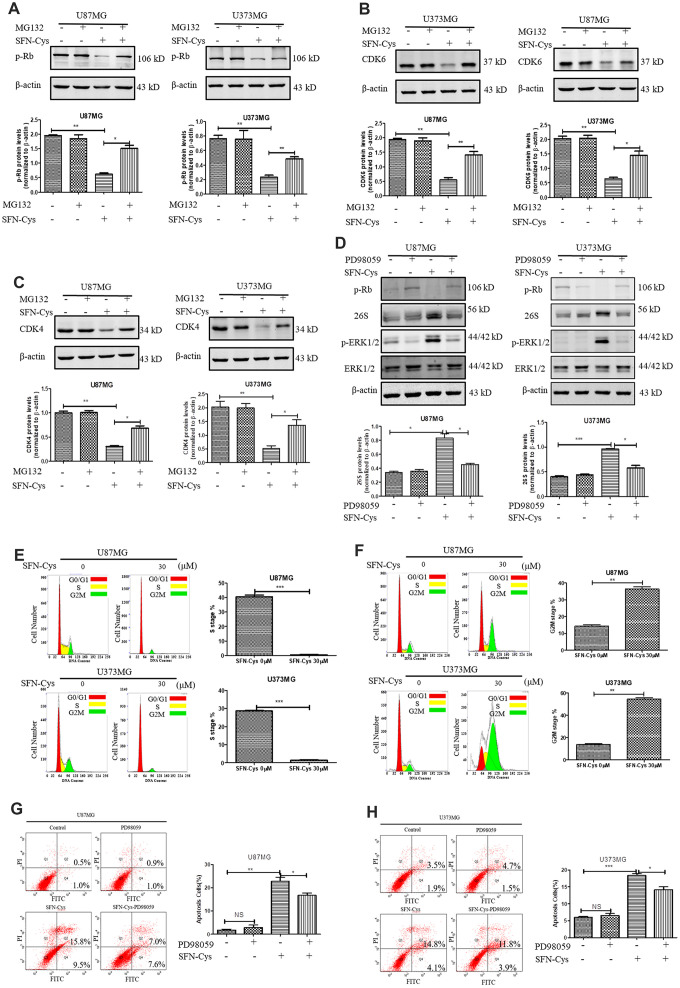
**SFN-Cys increased the expression of 26S proteasome via sustained ERK1/2 phosphorylation to degrade cell cycle-related proteins leading to the cell cycle arrest in G0/G1 and G2/M phases.** (**A**–**C**). The expressions of CDK4, CDK6 and p-Rb were detected by Western blot after the intervention of 30 μM SFN-Cys with or without MG132 (0.5 μM) for 24 h in U87MG and U373MG cells. (**D**) The expressions of 26S proteasome, ERK1/2 and p-ERK1/2 were detected by Western blot after the treatment of SFN-Cys (30 μM) with or without PD98059 (25 μM) for 24 h in U87MG and U373MG cells. (**E**) Both U87MG and U373MG cells were starved for 24 h, and then treated with or without SFN-Cys for 24 h. the cell cycles were detected by flow cytometry via Cell Cycle and Apoptosis Analysis Kit. (**F**) The cell cycles were detected without starvation. (**G**, **H**) The apoptosis rates were detected via Annexin V-FITC/PI staining in U87MG and U373MG cells after treatment of SFN-Cys (30 μM) with or without PD98059 (25 μM) for 24 h. The percentages of apoptotic cells were detected by flow cytometry. Data were shown as *P < 0.05; **P < 0.01; ***P < 0.001, NS no significance vs. control group, n ≥ 3.

The downregulation of CDK4/CDK6 and p-Rb by SFN-Cys was reversed after the addition of proteasome inhibitor MG132 (Figure 5A–5C). After SFN-Cys intervention, the expression of 26S proteasome was significantly upregulated; while ERK1/2 inhibitor PD98059 significantly reversed 26S proteasome upregulation (Figure 5D). Flow cytometry assay showed that the apoptosis rates in the U87MG and U373MG cells treated with SFN-Cys were significantly increased, and those effects were reversed by PD98059 (Figure 5G and 5H).

### SFN-Cys caused cell cycle arrest in G0/G1 and G2/M phases

In order to determine the effect of SFN-Cys on cell cycle, U87MG and U373MG cells were starved for 24 h in serum-free medium to synchronize cell cycle in G0/G1 phase; then cell cycle changes were detected by flow cytometry with a cell cycle detection kit after intervention with SFN-Cys for 24 h. We found that the cell numbers in the S phase were significantly reduced after the intervention with SFN-Cys and the cell cycle arrested in G0/G1 phase (Figure 5E). In order to clarify the influence of SFN-Cys on other cell cycle phases, these cells were directly intervened by SFN-Cys without synchronization for 24 h, followed by cell cycle detection. We found that after SFN-Cys intervention, the cell cycle was arrested in G2/M phase (Figure 5F).

### SFN-Cys caused cell cycle arrest in G2/M phase leading to apoptosis via microtubule disruption

**Figure 6:**
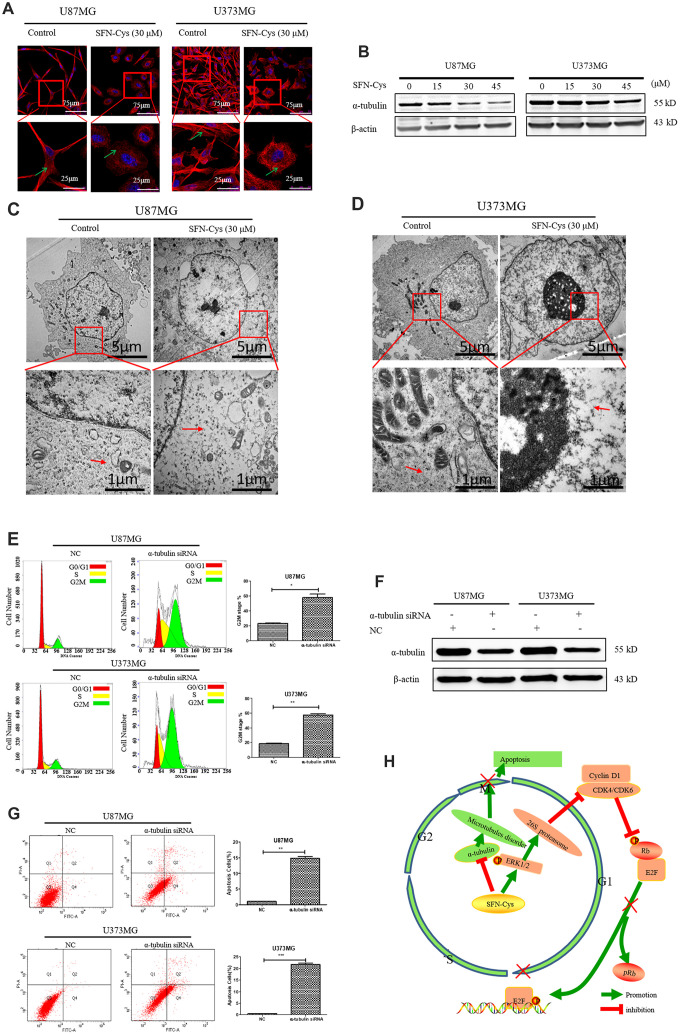
**The cell cycle was arrested in G2/M phase and apoptosis rate was significantly increased with the knockdown of α-tubulin; A possible signaling map was made for SFN-Cys-induced cell cycle arrest and apoptosis in human GBM cells.** (**A**) Immunofluorescence staining of α-tubulin showed the changes of microtubule morphology after treatment with or without SFN-Cys (30 μM) in U87MG/U373MG cells. Green arrows point to microtubules. (**B**) The expression of α-tubulin was detected by Western blot in U87MG and U373MG cells treated with or without SFN-Cys for 24 h. (**C**, **D**) The microtubules were observed via TEM in U87MG/U373MG cells treated with or without SFN-Cys (30 μM) for 24 h. Red arrows point to microtubules. (**E**, **G**) Both U87MG and U373MG cells were treated with α-tubulin siRNA for 72 h, and then the cell cycle and apoptosis rate were detected via flow cytometry. (**F**) The expression of α-tubulin was detected via Western blot after the intervention of α-tubulin siRNA for 72 h. (**H**) The possible signaling map for SFN-Cys-induced cell cycle arrest and apoptosis in human GBM cells.

Microtubule disorganization and aggregation were shown in both U87MG and U373MG cells treated with SFN-Cys for 24 h via immunofluorescence (Figure 6A). Further, we observed that SFN-Cys significantly downregulated the expressions of α-tubulin in a dose-dependent manner (Figure 6B). Furthermore, microtubule disruption and aggregation were observed via transmission electron microscopy (TEM) (Figure 6C and 6D). These implied that SFN-Cys disrupted microtubules in both cell lines. We also found that the cell cycle was arrested in G2/M phase and apoptosis rates were significantly increased after the knockdown of α-tubulin (Figure 6E and 6F). The possible signaling map was shown for SFN-Cys-induced cell cycle arrest and apoptosis in human GBM cells (Figure 6H).

## DISCUSSION

In the present studies, we demonstrated that SFN-Cys induced cell cycle arrest and apoptosis, which might result from microtubule disruption in GBM cells. Abnormal Rb regulation in tumor cells manifested with overexpressed CDK4/CDK6 and the loss of p16 expression or CDK4/CDK6 mutation leading to no response to p16 [33]. Here CDKN2A/CDKN2B deletion was frequent in GBM, which was consistent with previous report [12]. The P16, coded by CDKN2A/CDKN2B, was a main inhibitor of CDK4/CDK6; therefore, high deletion rate of CDKN2A/CDKN2B in GBM indicated that CDK4/CDK6 might be highly activated. Studies showed that CDK6, as an oncogene, was required for glioblastoma proliferation. Deletion of CDKN2A made cancer cells more sensitive to a selective activity inhibitor of CDK4/CDK6 [17]. The downregulation of CDK6 significantly inhibited cell viability and migration in human glioblastoma [34].

The activity inhibitors of CDK4/CDK6 had side effects and drug resistance [17, 35–37], thus SFN-Cys might be a candidate drug to target CDK4/CDK6.

SFN-Cys was a metabolite of SFN in vivo with lower toxicity [38]. We previously demonstrated that SFN-Cys had potentiality to inhibit proliferation and induce apoptosis in U87MG/U373MG cells [4], and its molecular mechanisms resulted from the activation of ERK1/2 and proteasome to degrade tumor-related proteins [18, 20, 21]. Studies showed that SFN-Cys was able to penetrate blood-brain barrier (BBB) with a longer half-life versus SFN in vivo [38], thus SFN-Cys might enter the central nervous cells through the blood-brain barrier and take action on GBM.

It was reported that SFN induced cell cycle arrest in ovarian epithelial tumors, breast cancer and other tumors [27, 28, 39], but its effect on GBM was not reported yet. Here we found that CDK4/CDK6 and TUBA1C were the main targets that SFN-Cys recognized via the mass spectrometry and gene ontology analysis. Overexpressions of CDK4/CDK6 and TUBA1C were predicted to high pathological grades and poor prognosis. Transient activation of ERK1/2 promoted cell proliferation and inhibited apoptosis [40], but sustained activation inhibited cell proliferation and induced apoptosis [41]. Our previous studies have found that SFN-Cys induced the sustained-activation of ERK1/2 [4, 21, 22]. Here we demonstrated that SFN-Cys significantly blocked the cell cycle in G0/G1 phase of U87MG/U373MG cells after cell cycle synchronization through starvation, and blocked the cell cycle in G2/M phase without synchronization. Mass spectrometry showed that SFN-Cys significantly reduced the expression of CDK4/CDK6 and TUBA1C. As we know, CDK4/CDK6 expression was correlated to cell cycle in G0/G1 phase; while TUBA1C expression was correlated to cell cycle in G2/M phase. Consequently, SFN-Cys-induced cell cycle arrest in G0/G1 phase and G2/M phase might be associated with the reduced expression of CDK4/CDK6 and TUBA1C.

We found that SFN-Cys significantly reduced the expressions of CDK4/CDK6 and p-Rb, and upregulated the expression of p-ERK1/2 in U87MG/U373MG cells. PD98059 reversed the downregulation of CDK4/CDK6 and p-Rb, suggesting that SFN-Cys induced the downregulation of CDK4/CDK6 through the sustained activation of ERK1/2, and the downregulation of CDK4/CDK6 resulted in decrease of p-Rb. While the expression of Cyclin D1 increased, the co-localization of CDK4/CDK6 to Cyclin D1 was decreased; these might result from the downregulation of CDK4/CDK6 in the cells treated with SFN-Cys. The decreased p-Rb caused reducing release of E2F into nucleus, resulting in cell cycle arrest in G0/G1 phase.

It was reported that tumor cells were blocked in G0/G1 phase due to the activation of proteasome; while the proliferation ability was significantly increased after the intervention of proteasome inhibitor [23, 25]. We previously reported that SFN-Cys might activate 26S proteasome in human non-small cell lung cancer cells [18, 20], thus CDK4/CDK6 might be degraded by the ubiquitin-proteasome pathway. In this study, it was found that the proteasome inhibitor MG132 reversed the downregulation of CDK4/CDK6 induced by SFN-Cys in U87MG and U373MG cells, indicating that SFN-Cys decreased the expression of CDK4/CDK6 by activating proteasome. Meanwhile, SFN-Cys significantly upregulated the expression of 26S proteasome; the upregulation could be reversed by PD98059. The results demonstrated that SFN-Cys increased the expression of 26S proteasome via p-ERK1/2. These data indicated that SFN-Cys decreased the expression of CDK4/CDK6 by sustained ERK1/2 phosphorylation to activate 26S proteasome; the decrease of CDK4/CDK6 contributed to cell cycle arrest in G0/G1 phase.

Reports showed that microtubules were the important targets in the treatment of glioblastoma, and microtubules disruption significantly caused apoptosis [30–32]. The microtubule-targeting drug paclitaxel significantly inhibited the proliferation, migration, and invasion in U87MG cells [42]. Microtubule targeting is one of the main mechanisms in fighting cancer [43]. We previously reported that SFN-mediated upregulation of 26S proteasome via sustained ERK1/2 phosphorylation leading to microtubule disruption and cell apoptosis [20, 21]. Here we further found that SFN-Cys downregulated α-tubulin, disrupted microtubules and induced cell cycle arrest in G2/M phase. We also found that the cell cycle was arrested in G2/M phase and apoptosis rate was significantly increased with the knockdown of α-tubulin. These indicated that SFN-Cys-induced microtubules disruption contributed to cell cycle arrest and apoptosis in U87MG and U373MG cells.

Studies showed that CDK4/CDK6 inhibition promoted apoptosis in tumor cells [15]. After SFN-Cys intervention, cell apoptosis rates were significantly increased; PD98059 reversed the increase. Therefore, SFN-Cys might induce apoptosis via activation of ERK1/2 and 26S proteasome to degrade CDK4/CDK6.

CDK4/CDK6 inhibitors might not be fully effective for the treatment of recurrent GBM [44]. SFN-Cys might work only or combine with the current CDK4/CDK6 inhibitors to fight GBM.

In conclusion, these studies showed that SFN-Cys downregulated CDK4/CDK6 by phosphorylated ERK1/2-caused proteasomal degradation resulting in decreased Rb phosphorylation and thus contributing to cell cycle arrest in G0/G1 phase. Moreover, SFN-Cys caused cell cycle arrest in G2/M phase leading to apoptosis, which might result from microtubule disruption (Figure 6H). These results provided a strong theoretical basis for us to use SFN-Cys as a potential drug to treat GBM patients.

## MATERIALS AND METHODS

### Antibodies and reagents

D, L-Sulforaphane-L-cysteine (SFN-Cys) was bought from Santa Cruz Biotechnology (Dallas, TX, USA). Anti-β-actin, anti-α-tubulin and anti-Cyclin D1 were purchased from Santa Cruz Biotechnology (Dallas, TX, USA). Anti-CDK4, anti-CDK6, anti-Rb and anti-p-Rb (S780) were purchased from Abcam (Cambridge, UK). Anti-ERK1/2 and anti-p-ERK1/2 (Thr202/Tyr204) and PD98059 were obtained from Cell Signaling Technology Inc (Danvers, MA, USA). Annexin V-FITC Apoptosis assay kit was acquired from Genstar (Beijing, China). Cell Cycle and Apoptosis Analysis Kit was obtained from Beyotime Biotechnology (Shanghai, China).

### Cell culture

Human glioblastoma U87MG cell line was obtained from the Cell Resource Center, Peking Union Medical College (CRC/PUMC) and U373MG cell line was obtained from American Type Culture Collection (ATCC, Manassas, VA, USA). Cells were cultured in DMEM/HIGH glucose culture medium supplemented with 10% FBS, in a standard humidified incubator with 5% CO_2_ at 37°C. The cells were treated with SFN-Cys for 24 h and pretreated with p-ERK1/2 inhibitor PD98059 (25 μM) or proteasome inhibitor MG132 (0.5 μM) for 30 min.

### High performance liquid chromatography-mass spectrometry/mass spectrometry analysis

High Performance Liquid Chromatography-mass Spectrometry/Mass Spectrometry (HPLC-MS/MS) was used to analyze the protein expressions in U87MG cells. Cell lysates were harvested and quantitated after treated with or without SFN-Cys for 24 h. Equal amount of protein was analyzed by HPLC-MS/MS. After the data were identified, protein expressions were analyzed by the Uniprot net for cell localization and function variations.

### Bioinformatics analysis

In the Chinese Glioma Genome Atlas (CGGA) databases, we selected the mRNA expression data in the 693 samples, ranging from WHO grade II to grade IV, generated by Agilent Whole Human Genome Array Platform [45]. These results have been obtained through the tool provided in the CGGA. In TCGA and GTEx databases [46], we searched the GEPIA (Gene Expression Profiling Interactive Analysis) Database to find the possible correlation of survival rate to expressions of α-tubulin, CDK6, CDK4 and recorded these results. These data for gene deletion in glioblastomas were obtained through cBioPortal with the Glioblastoma Multiforme (TCGA, Provisional) and Merged Cohort of LGG and GBM (TCGA, Cell 2016).

### Transmission electron microscopy (TEM)

The treated cells were harvested and fixed with cold glutaraldehyde (3%) and osmic acid (1%), and then the cell pellet was embedded in epoxy resin and sectioned for TEM observation.

### Immunofluorescence and confocal microscopy

The treated cells were fixed with 1% paraformaldehyde for 15 min and permeabilized with methanol −20°C for 10 min. After blocking by 5% BSA for 1h at room temperature, the cells were incubated with the corresponding primary antibodies overnight at 4 °C and further incubated with the fluorescence-labeled secondary antibody 1 h at room temperature. The cells were stained with DAPI and observed under a laser scanning confocal microscope (LSCM) (Olympus FV1000, Japan).

### Western blot analysis

Cells were lysed with RIPA and protein concentrations were tested by BCA protein assay kit. Proteins were separated on SDS-PAGE gels and then transferred to nitrocellulose membranes. Membranes were blocked with 1.5% BSA in TBS Tween-20 (TBS-T) buffer for 1h at room temperature and then incubated with primary antibody at 4°C overnight. Then the membrane was washed for three times with TBS-T and incubated with the fluorescence-labeled secondary antibody. Protein bands were detected by Odyssey infrared imaging system (LI-COR Biosciences, Lincoln, NE, USA).

### Cell transfection

In order to knockdown α-tubulin, negative control siRNA (NC siRNA) and α-tubulin siRNA (5′-AGAUGUCAAUGCUGCCAUU-3′) were designed [47]. Cells were plated in 6-well plates (diameter, 35 mm) at a density of 1×10^6^/well and cultured for 24 h. Then the cells were transfected with the siRNA or NC siRNA (30 pmol/well) by Lipofectamine™ RNAiMAX (Invitrogen, USA) for 72h, then the transfected cells were used for the cell cycle and apoptosis assay.

### Cell cycle assay

Cells were divided into two groups. One group of cells was starved for 24 h in serum-free medium to synchronize the cell cycle. The other group of cells was not starved. Two groups were treated with or without SFN-Cys (30 μM) for 24 h. Then these cells were detached by trypsinization, washed with PBS for three times, and fixed in 70% ethanol at 4 °C for 24 h. Next, these cells were washed with phosphate-buffered saline (PBS) and stained with 50 μg/ml PI at 37 °C for 30 min. PI fluorescence was measured by a flow cytometer (BD Biosciences, Rutherford, NJ).

### Apoptosis detection

Cells were treated with SFN-Cys (30 μM) with or without PD98059 (25 μM) for 24 h and then the adhesive cells were collected and washed twice with cold PBS. Next, the cells were re-suspended at a concentration of 1×10^6^ cells/ml in binding buffer and incubated with annexin V-FITC for 5 min and PI was added afterwards in the dark. Cells were analyzed by the flow cytometer (BD Biosciences, Rutherford, NJ).

### Statistical analysis

All data were shown as mean ± standard deviation (SD) from 3 independent experiments. Paired data were evaluated by Mann Whitney test and multiple independent data by Kruskal-Wallis test. P≤0.05 was considered statistically significant. The statistical analyses were done by SPSS version 19.0.
